# A Two-Stage Meta-Analysis Identifies Several New Loci for Parkinson's Disease

**DOI:** 10.1371/journal.pgen.1002142

**Published:** 2011-06-30

**Authors:** International Parkinson's Disease Genomics Consortium (IPDGC), Wellcome Trust Case Control Consortium 2 (WTCCC2)

**Affiliations:** Georgia Institute of Technology, United States of America

## Abstract

A previous genome-wide association (GWA) meta-analysis of 12,386 PD cases and 21,026 controls conducted by the International Parkinson's Disease Genomics Consortium (IPDGC) discovered or confirmed 11 Parkinson's disease (PD) loci. This first analysis of the two-stage IPDGC study focused on the set of loci that passed genome-wide significance in the first stage GWA scan. However, the second stage genotyping array, the ImmunoChip, included a larger set of 1,920 SNPs selected on the basis of the GWA analysis. Here, we analyzed this set of 1,920 SNPs, and we identified five additional PD risk loci (combined *p<5*×*10^−10^*, *PARK16*/1q32, *STX1B*/16p11, *FGF20*/8p22, *STBD1*/4q21, and *GPNMB*/7p15). Two of these five loci have been suggested by previous association studies (*PARK16*/1q32, *FGF20*/8p22), and this study provides further support for these findings. Using a dataset of post-mortem brain samples assayed for gene expression (n = 399) and methylation (n = 292), we identified methylation and expression changes associated with PD risk variants in *PARK16*/1q32, *GPNMB*/7p15, and *STX1B*/16p11 loci, hence suggesting potential molecular mechanisms and candidate genes at these risk loci.

## Author Summary

This paper describes the largest case-control analysis of Parkinson's disease to date, with a combined sample set of over 12,000 cases and 21,000 controls. After combining our findings with an independent replication dataset of more than 3,000 cases and 29,000 controls, we found five additional PD risk loci in addition to the 11 loci previously identified in earlier consortium efforts. This successful study further demonstrates the power of the GWA scan experimental design to find new loci contributing to disease risk, even in the context of complex disorders like Parkinson's disease. These new findings provide insights into the etiology of PD and will promote a better understanding of its pathogenesis.

## Introduction

Until the recent developments of high throughput genotyping and genome-wide association (GWA) studies, little was known of the genetics of typical Parkinson's disease (PD). Studies of the genetic basis of familial forms of PD first identified rare highly penetrant mutations in *LRKK2* [1], [2], *PINK1* [3], *SNCA* [4], *PARK2* [5] and *PARK7* [6]. Following these findings, GWA scans for idiopathic PD identified *SNCA* and *MAPT* as unequivocal risk loci [7], [8], [9], [10], [11] as well as implicated *BST1* [8], *GAK* [12], and *HLA-DR* [13]. Using sequence based imputation methods [14], the meta-analysis of several GWA scans [7], [9], [10], [11] conducted by the International Parkinson's Disease Genomics Consortium (IPDGC) identified and replicated five new loci: *ACMSD*, *STK39*, *MCCC1*/*LAMP3*, *SYT11*, and *CCDC62*/*HIP1R* [15] and confirmed association at *SNCA*, *LRRK2*, *MAPT*, *BST1*, *GAK* and *HLA-DR* [15].

We conducted a two-stage association study. Combining stage 1 and stage 2, the data consist of 12,386 PD cases and 21,026 controls genotyped using a variety of platforms (Table 1). Stage 1 used genome-wide genotyping arrays and our initial analysis [15] focused on the subset of SNPs that passed genome-wide significance in stage 1. For stage 2 genotyping, we used a custom content Illumina iSelect array, the ImmunoChip and additional GWAS typing as previously described [15]. The primary content of the ImmunoChip data focuses on autoimmune disorders but, as part of a collaborative agreement with the Wellcome Trust Case Control Consortium 2, we included 1,920 ImmunoChip SNPs on the basis of the stage 1 GWA PD results.

**Table 1:** Sample size and genotyping platform for the cohorts included in stage 1 (top set of rows), stage 2 (middle set of rows), and independent replication (bottom row).

Cohort	Controls	Cases	Genotyping platform
United Kingdom	5,200	1,705	Illumina 660W-Quad
USA-NIA	3,034	971	Illumina HumanHap 550
USA-dbGAP	857	876	Illumina 370 K
German	944	742	Illumina HumanHap550
French	1,984	1039	Illumina 610-Quad
**Total Stage 1**	**12,019**	**5,333**	
Icelandic	1,427	479	Illumina HumanHap 300
Dutch	2,024	772	Illumina 610-Quad
USA	2,215	2,807	ImmunoChip
United Kingdom	1,864	1,271	ImmunoChip
Dutch	402	304	ImmunoChip
French	363	267	ImmunoChip
German	712	1,153	ImmunoChip
**Total Stage 2**	**9,007**	**7,053**	
**Stage 1+Stage 2**	**21,026**	**12,386**	
**Do et al- USA**	**29,624**	**3,426**	

Here, we report the combined analysis for this full set of 1,920 SNPs. This step1+2 analysis identified seven new loci that passed genome-wide significance in the meta-analysis. During the process of analyzing these data and preparing for publication, we became aware that another group was also preparing a large independent GWA scan in PD for publication (Do et al, submitted). Following discussion with this group we agreed to cross validate the top hits from each study by exchanging summary statistics for this small number of loci.

To provide further insights into the molecular function of these associated variants, we tested risk alleles at these loci for correlation with the expression of physically close gene (expression quantitative trait locus, eQTL) and the methylation status (methQTL) of proximal DNA CpG sites in a dataset of 399 control frontal cortex and cerebellar tissue samples extracted post-mortem from individuals without a history of neurological disorders.

## Results

In addition to eleven loci that passed genome-wide significance in stage 1 [15], we identified over 100 regions of interest defined as 10 kb windows containing at least one SNP associated at *p*<10^−3^. We submitted the most associated SNP in each region for probe design and follow-up genotyping using the ImmunoChip platform. For each region of interest, we also added four SNPs in high level of linkage disequilibrium (LD) to provide redundancy where the most associated SNP would not pass the Illumina probe design step or the assay for that SNP would fail. To complete the array design we also added all non-synonymous dbSNPs located in known PD associated regions [1], [2], [3], [4], [5], [6]. Out of these 2,400 submitted SNPs, 1,920 passed QC and were included in the final array design. For these 1,920 SNPs we combined stage 1 and stage 2 associated data in a meta-analysis of 12,386 cases and 21,026 controls (Table 1) from the IPDGC. We exchanged summary statistics for these most significant hits with an additional large, case-control replication dataset (3,426 PD cases and 29,624 controls) in an attempt to demonstrate independent replication.

**Figure 1:**
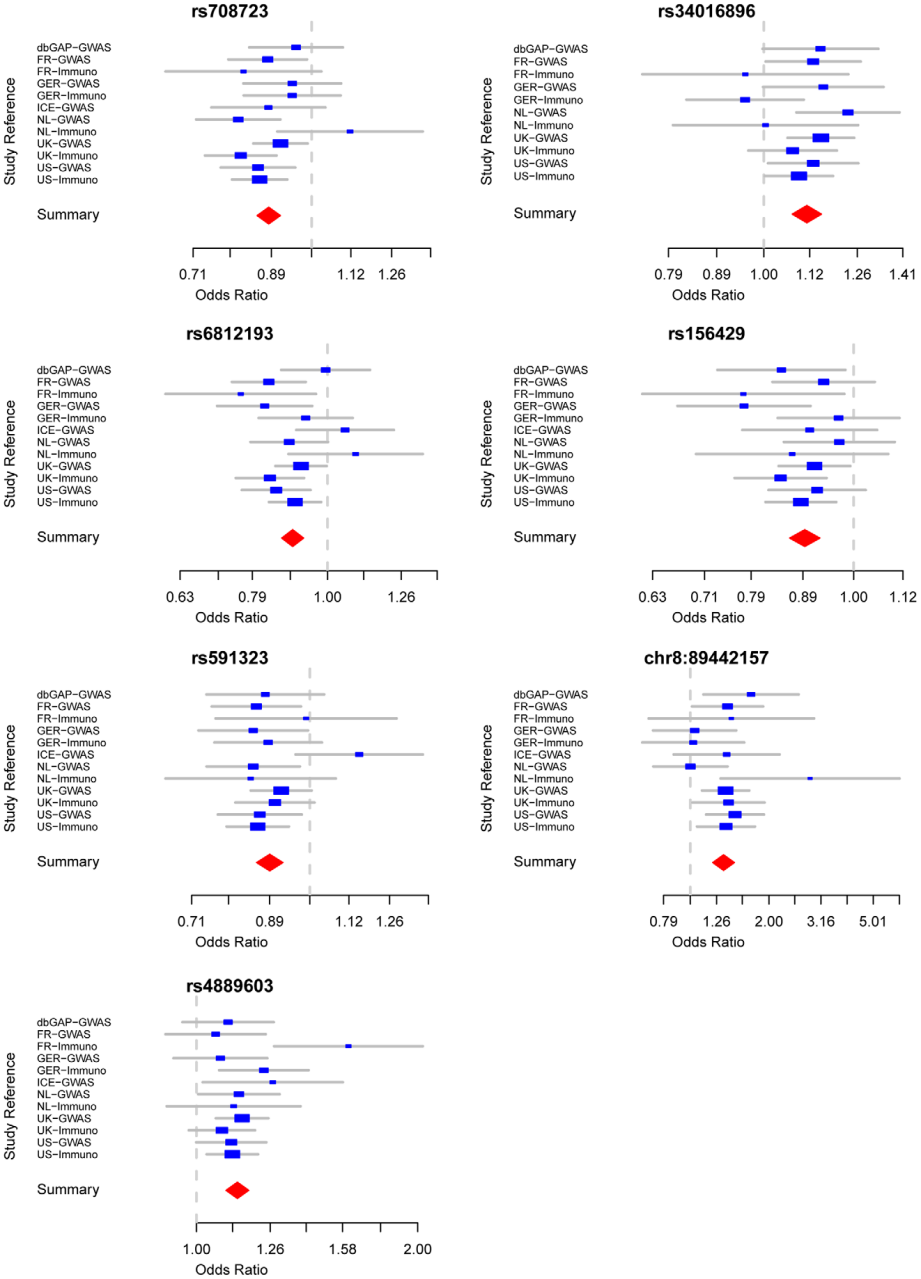
Forest plots detailing effect estimates from the combined analysis of all data contributed by the International Parkinson Disease Genomics Consortium (joint estimates describing constituent effects of Stage 1+Stage 2).

On the basis of stage 1+2 results, seven new SNPs passed our defined genome-wide significance threshold (*p*<5×10^−8^, Table 2 and Figure 1). These loci are either novel or the previous evidence of association was not entirely convincing in individuals of European descent. We combined these results with the independent replication. Five of these seven loci replicated and showed strong combined evidence of PD association (p<10^−10^ overall). Taking either the nearest gene (or the strongest candidate when available) to designate these regions, these five loci are 1q32/*PARK16* [7], 4q21/*STBD1*, 7p15/*GPNMB*, 8p22/*FGF20* [16] and 16p11/*STX1B*.

**Table 2:** Summary statistics for the seven SNPs that pass genome-wide significance (*p*<5×10^−8^) in the combined stage 1+2 analysis and that have either not been reported in published PD association studies.

					Stage 1		Stage 2		Stage 1+2	Do et al		Combined
SNP	Chrom	Gene(s)	Alleles	MAF	OR (95%CI)	*P*	OR (95%CI)	*P*	*P*	OR (95%CI)	*P*	*P*
rs708723	1q32	*RAB7L1/PARK16*	T>C	0.439	0.905 (0.862–0.95)	6.68×10^−5^	0.863 (0.824–0.905)	9.47×10^−10^	1.00×10^−12^	0.758 (0.65–0.88)	2.12×10^−6^	8.82×10^−15^
rs34016896	3q26	*NMD3*	C>T	0.305	1.14 (1.09–1.2)	3.00×10^−7^	1.08 (1.02–1.14)	0.00399	1.81×10^−8^	1.002 (0.95–1.06)	0.954	1.31×10^−6^
rs6812193	4q21	*STBD1*	C>T	0.36	0.886 (0.843–0.932)	2.52×10^−6^	0.906 (0.864–0.95)	5.29×10^−5^	7.46×10^−10^	0.839 (0.79–0.89)	7.55×10^−10^	1.17×10^−17^
rs156429	7p15	*GPNMB*	A>G	0.403	0.894 (0.849–0.942)	2.15×10^−5^	0.893 (0.852–0.937)	3.86×10^−6^	3.27×10^−10^	0.901 (0.85–0.95)	0.000193	3.05×10^−13^
rs591323	8p22	*FGF20*	G>A	0.271	0.884 (0.836–0.935)	1.59×10^−5^	0.875 (0.83–0.923)	8.49E×10^−7^	7.45×10^−11^	0.932 (0.88–0.99)	0.023	1.92×10^−11^
chr8:89442157	8q21	*MMP16*	C>T	0.0247	1.38 (1.21–1.57)	1.10×10^−6^	1.29 (1.12–1.49)	0.000451	2.26×10^−9^	0.969 (0.86–1.09)	0.589	2.36×10^−5^
rs4889603	16p11	*STX1B*	A>G	0.413	1.12 (1.06–1.18)	4.13×10^−5^	1.15 (1.1–1.21)	8.21×10^−9^	2.66×10^−12^	1.070 (1.01–1.13)	0.014	6.98×10^−13^

1q32/PARK16 has been reported previously but is included because these data provide for the first time unequivocal evidence of association. P-values are computed using a one-degree-of-freedom regression trend test, including two principal components as covariates and combining the results across cohorts using a score test methodology. P-values are two-tailed and odds ratios are reported for the minor alleles. The notation X>Y indicates that X is the major allele and Y the minor allele. Allele frequencies were estimated using the UK control data. OR: odds ratio.

rs708723/1q32 has been previously reported as PD associated (*PARK16*, [7], [8]) but this SNP lacked the unequivocal evidence of association in European samples (*p* = 9.47×10^−10^ in stage 2 only). To understand the potential biological consequences of risk variation at this locus we tested whether rs708723 was correlated with either gene expression or DNA methylation status of proximal transcripts or CpG sites respectively (Table 3). We found correlations with the expression of *NUCKS1* (*p* = 1.8×10^−7^) and *RAB7L1* (*p* = 7.2×10^−4^). We also found correlations with the methylation state of CpG sites located in the *FLJ3269* gene (*p* = 3.9×10^−22^).

**Table 3:** Significant eQTL associations (*p*<0.01) between the five SNPs with positive replication data (Table 2) and proximal (cis) changes in gene expression/methylation in frontal cortex and cerebellar tissue.

Assay	Region	SNP	Region	Gene Tagged by Probe	Illumina Probe	Alleles	Effect Estimate	Standard Error	Unadjusted P	False Discovery Rate Adjusted P
Expression	Frontal Cortex	rs156429	7p15/*GPNMB*	*NUPL2*	ILMN_1789616	A>G	0.083	0.018	3.6E-06	1.0E-04
		rs156429	7p15/*GPNMB*	*NUPL2*	ILMN_2115154	A>G	0.078	0.017	3.1E-06	1.0E-04
		rs708723	1q32/*PARK16*	*NUCKS1*	ILMN_1680692	T>C	0.155	0.03	1.8E-07	1.5E-05
		rs708723	1q32/*PARK16*	*RAB7L1*	ILMN_1813685	T>C	−0.062	0.018	7.2E-04	1.2E-02
		rs4889603	16p11/*STX1B*	*ZNF668*	ILMN_1739236	A>G	0.062	0.015	4.1E-05	8.7E-04
		rs4889603	16p11/*STX1B*	*MYST1*	ILMN_1804679	A>G	−0.053	0.018	3.4E-03	4.8E-02
	Cerebellum	rs156429	7p15/*GPNMB*	*NUPL2*	ILMN_1789616	A>G	0.133	0.025	1.0E-07	3.7E-06
		rs156429	7p15/*GPNMB*	*NUPL2*	ILMN_2115154	A>G	0.131	0.023	1.2E-08	1.0E-06
		rs708723	1q32/*PARK16*	*NUCKS1*	ILMN_1680692	T>C	0.13	0.029	5.3E-06	1.1E-04
		rs708723	1q32/*PARK16*	*RAB7L1*	ILMN_1813685	T>C	−0.106	0.02	1.3E-07	3.7E-06
		rs4889603	16p11/*STX1B*	*ZNF668*	ILMN_1739236	A>G	0.075	0.02	1.3E-04	2.3E-03
		rs4889603	16p11/*STX1B*	*BCL7C*	ILMN_2371147	A>G	0.066	0.022	2.6E-03	3.8E-02
Methylation	Frontal Cortex	rs156429	7p15/*GPNMB*	*GPNMB*	cg17274742	A>G	−0.027	0.005	5.1E-07	3.2E-05
		rs156429	7p15/*GPNMB*	*GPNMB*	cg22932819	A>G	−0.009	0.002	1.6E-07	1.3E-05
		rs6812193	4q21/*STBD1*	*GENX-3414*	cg17010112	C>T	0.008	0.002	9.4E-04	3.0E-02
		rs708723	1q32/*PARK16*	*FLJ32569*	cg14159672	T>C	−0.219	0.022	3.1E-24	3.9E-22
		rs708723	1q32/*PARK16*	*FLJ32569*	cg14893161	T>C	−0.176	0.017	3.9E-25	9.6E-23
		rs4889603	16p11/*STX1B*	*BCL7C*	cg07896225	A>G	−0.002	0.001	9.7E-04	3.0E-02
		rs4889603	16p11/*STX1B*	*STX1B*	cg25033993	A>G	0.012	0.003	8.2E-05	3.4E-03
	Cerebellum	rs156429	7p15/*GPNMB*	*GPNMB*	cg17274742	A>G	−0.015	0.003	2.1E-06	1.3E-04
		rs708723	1q32/*PARK16*	*FLJ32569*	cg14159672	T>C	−0.246	0.023	3.0E-27	3.7E-25
		rs708723	1q32/*PARK16*	*FLJ32569*	cg14893161	T>C	−0.202	0.018	2.6E-28	6.4E-26

In the case of 16p11/*STX1B*, the proximal gene to the most associated SNP rs4889603 is *SETD1A*. However, *STX1B* is located 18 kb upstream of rs4889603 and is a more plausible PD candidate gene [17] owing to its synaptic receptor function. We therefore used this gene to designate this region. Our methQTL/eQTL dataset identified a correlation between the rs4889603 risk allele and increased methylation of a CpG dinucleotide in *STX1B* (Table 3).

The SNP rs591323 in the 8p22 region is located ∼150 kb downstream of the *FGF20* gene (NCBI build 36.3), for which association with PD has been suggested previously in familial PD samples [16], [18] but which remained controversial [19]. Our findings provide further support for a PD association at this locus, but again, whether the functionally affected transcript is *FGF20* or not remains unclear.

The regions 4q21/*STBD1* and 7p15/*GPNMD* have not been previously implicated in PD etiology. We found that the risk allele of rs156429, the most associated SNP in the 7p15 region, is associated in our eQTL dataset with decreased expression of the proximal transcript encoded by *NUPL2* (Table 3). The same risk allele is also associated with increased methylation of multiple CpG sites proximal to *GPNMB* itself (Table 3). Neither of these regions contains an obvious candidate gene.

Two additional loci (3q26/*NMD3* and 8q21/*MMP16*) showed strong evidence of association in stage 1 and 2 but were not disease associated in the Do et al dataset. Further replication is required to clarify the role of variation at these loci in risk for PD.

The strongly associated G2019S variant in the *LRRK2* gene [20] was included in the Immunochip design and we replicated the published association: control frequency: 0.045% case frequency 0.61%, estimated odds ratio: 13.5 with 95% confidence interval: 5.5–43. However, the case collections have been partially screened for this variant therefore its frequency in cases and the odds ratio is likely to be underestimated.

The ImmunoChip array design provides some power to detect whether multiple distinct association signals exist at individual loci. Indeed, if a SNP showed an independent and sufficiently strong association in stage 1, it would have been included in stage 2 provided that it was not located in the same 10 kb window as the primary SNP in the region. There is precedent for this in PD, with the previous identification of independent risk signals at the *SNCA* locus [11]. We therefore used the Immunochip data to test whether any of the seven loci in Table 2 showed some evidence of more than one independent signal. None of these seven loci showed any association (*p*>0.01) after conditioning on the main SNP in the region. In contrast, after conditioning on the most associated SNPs rs356182 in the *SNCA* region, several SNPs remained convincingly associated (*p* = 9.7×10^−8^ for rs2245801 being the most significant).

Lastly, we performed a risk profile analysis to investigate the power to discriminate cases and controls on the basis of the 16 confirmed common associated variants (Table 4). For each locus, we estimated the odds ratio on the basis of stage 1 data and we applied these estimates to compute for each individual in the ImmunoChip cohort a combined risk score. Solely based on these 16 common variants, and therefore not considering rare highly penetrant variants such as G2019S in *LRKK2* [20], we found that individuals in the top quintile of the risk score have an estimated three-fold increase in PD risk compared to individuals in the bottom quintile (Table 4). We note however that the effect size of several of these associated variants could be over-estimated (an effect known as winner's curse, see [21]) but given the consistent estimates of odds ratio across studies (Table 4) we expect this bias to be minimal.

**Table 4:** Estimated PD risk profile for the five cohorts genotyped using the Immunochip.

			*1^st^ quintile*		*2^nd^ quintile*		*3^rd^ quintile*		*4^th^ quintile*		*5^th^ quintile*	
*Study*	*Trend P-value*	*AUC*	*OR*	*95% CI*	*OR*	*95% CI*	*OR*	*95% CI*	*OR*	*95% CI*	*OR*	*95% CI*
USA	<2E-16	0.614	1	–	1.54	1.29–1.84	1.92	1.61–2.29	2.21	1.85–2.65	3.03	2.52–3.64
UK	<2e-16	0.636	1	–	1.34	1.05–1.71	1.79	1.41–2.28	2.35	1.86–2.99	3.11	2.46–3.96
Germany	1.29E-11	0.692	1	–	1.32	0.98–1.79	1.88	1.38–2.58	1.88	1.38–2.56	2.57	1.88–3.53
France	5.19E-13	0.675	1	–	1.69	0.99–2.92	1.13	0.65–1.98	3.30	1.95–5.67	5.92	3.42–10.52
Netherlands	5.08E-05	0.601	1	–	1.06	0.65–1.74	1.35	0.83–2.20	1.91	1.18–3.11	2.36	1.45–3.86
Combined	<2E-16	0.645	1	–	1.43	1.26–1.61	1.79	1.58–2.02	2.22	1.96–2.50	3.02	2.67–3.42
**% Cases per Quintile**				**37.90**		**46.06**		**51.15**		**56.56**		**63.75**

Risk scores for the 16 confirmed loci were computed using the odds ratio estimated from the genome-wide case-control genotype data. Individuals were split into quintile on the basis of their risk scores. The odds ratios quantify the effect of the computed risk quintile on the probability of being a PD case (one-degree-of-freedom logistic trend test with the PD status as a binary outcome variable and the quintiles, coded as 1–5, as covariates). The first quantile group was taken as a reference group. OR: odds ratio, CI: confidence interval.

## Discussion

The combination of GWA scans and imputation methods in large cohorts of PD cases and controls has enabled us to identify five PD associated loci in addition to the 11 previously reported by us. Two of these loci (1q32/*PARK16*, 8p22/*FGF20*) implicate regions that had been previously associated with PD risk [8], [16]. The 1q32/*PARK16* showed convincing evidence of association in the Japanese population [8] but until now the association P-value had not passed a stringent genome-wide significance threshold in samples of European descent [7]. The 8p22/*FGF20* locus had been previously reported in a study of familial PD [16] and we provide the first evidence of association in a case-control study. The remaining three loci (*STX1B*/16p11, *STBD1*/4q21 and *GPNMB*/7p15) are new.

Adding the eleven previously reported common variants [15] to the five convincingly associated loci identified in this study, common variants at 16 loci have now been associated with PD. Controlling for the risk score based on the 11 SNPs previously identified [15] in the risk profile analysis (Table 4), the addition of these five new loci provides a modest but significant (*p* = 2.2×10^−3^) improvement of our ability to discriminate PD cases from controls.

Combining eQTL/methylation and case-control data implicates potential mechanisms which could explain the increased PD risk associated some of these variants. In particular, the strong eQTL in the 1q32/*PARK16* region with the *RAB7L1* and *NUCKS1* genes (Table 3) suggests that either one of these genes could be the biological effector of this risk locus. However, existing data show that eQTLs are widespread and this co-localization could be the result of chance alone [22]. Additional fine-mapping work will be required to assess whether the expression and case-control data are indeed fully consistent.

While we are unable to unequivocally pinpoint the causative genes underlying these associations, their known biological function can suggest likely candidates. At the 1q32/*PARK16* loci our association and eQTL data indicate that *RAB7L1* and *NUCKS1* are the best candidates. The former is a GTP-binding protein that plays an important role in the regulation of exocytotic and endocytotic pathways [23]. Exocytosis is relevant for PD for two main reasons: firstly, since dopaminergic neurotransmission is mediated by the vesicular release of dopamine, i.e. dopamine exocytosis [24], and secondly because it has been shown that alpha-synuclein knock-out mice develop vesicle abnormalities [25], thus providing a potential direct link between genetic variability in the gene and a biological pathway involved in the disease. Less is known regarding *NUCKS1*; it has been described to be a nuclear protein, containing casein kinase II and cyclin-dependant kinases phosphorylation sites and to be highly expressed in the cardiac muscle [26]; but an involvement in PD pathogenesis has yet to be suggested.

At the 16p11/*STX1B* locus, notwithstanding the fact that other genes are in the associated region, *STX1B* is the most plausible candidate. It has been previously shown to be directly implicated in the process of calcium-dependent synaptic transmission in rat brain [17], having been suggested to play a role in the excitatory pathway of synaptic transmission. Since parkin, encoded by *PARK2*, negatively regulates the number and strength of excitatory synapses [27] , it makes *STX1B* a very interesting candidate from a biologic perspective.

*FGF20* at 8p22 has been suggested to be involved in PD [16], albeit negative results in smaller cohorts have followed the original finding [28]. *FGF20* is a neurotrophic factor that exerts strong neurotrophic properties within brain tissue, and regulates central nervous development and function [29]. It is preferentially expressed in the substantia nigra [30], and it has been reported to be involved in dopaminergic neurons survival [30].

The ImmunoChip data provide limited resolution for the detection of multiple independent association signals in these regions. A previous study [31] reported some evidence of allelic heterogeneity at the 1q32/*PARK16* locus but the ImmunoChip data do not support this result. A previous study [11] also reported two independent associations at the 4q22/*SNCA* locus and our data are consistent with this scenario. However, the newly reported secondary association (rs2245801) is in low LD (r^2^ = 0.21) with rs2301134, the SNP reported in [11] as an independent association. Taken together, these findings suggest that at least three independent associations exist at *SNCA*/4q22. A more exhaustive fine-mapping analysis using either sequencing of large cohorts or targeted genotyping arrays will also be required to fully explore this locus.

As yet, we do not know which of the variants and which genes within each region are exerting the pathogenic effect. We cannot exclude that some of the currently reported variants are in fact tagging high penetrance, but rare, mutations [32]. Nevertheless, the successful identification of these 16 risk loci further demonstrates the power of the GWA study design, even in the context of disorders like PD that have a complex genetic component. We therefore expect that further and larger association analyses, perhaps using dedicated high-throughput genotyping arrays like the ImmunoChip, will continue to yield new insights into PD etiology.

## Material and Methods

### Genotyping and case control cohorts

Participating studies were either genotyped using the ImmunoChip as part of a collaborative agreement with the ImmunoChip Consortium, or as part of previous GWA studies provided by members of the IPDGC or freely available from dbGaP [7], [9], [10], [11]. Genotyping of the UK cases using the Immunochip was undertaken by the WTCCC2 at the Wellcome Trust Sanger Institute which also genotyped the UK control samples. The constituent studies comprising the IPDGC have been described in detail elsewhere [15], although a summary of individual study quality control is available as part of Table S1. In brief all studies followed relatively uniform quality control procedures such as: minimum call rate per sample of 95%, mandatory concordance between self-reported and X-chromosome-heterogeneity estimated sex, exclusion of SNPs with greater than 5% missingness, Hardy Weinberg equilibrium p-values at a minimum of 10^−7^, minor allele frequencies at a minimum of 1%, exclusion of first degree relatives, and the exclusion of ancestry outliers based on either principal components or multidimensional scaling analyses using either PLINK [33] or EIGENSTRAT [34] to remove non-European ancestry samples. All GWAS studies utilized in this analysis (and in the QTL analyses) were imputed using MACHv1.0.16 [14] to conduct a two-stage imputation based on the August 2009 haplotypes from initial low coverage sequencing of 112 European ancestry samples in the 1000 Genomes Project [35], filtering the data for a minimum imputation quality of (RSQR>0.3) [14]. Logistic regression models were utilized to quantify associations with PD incorporating allele dosages as the primary predictor of disease. Imputed data was analyzed using MACH2DAT, and genotyped SNPs were analyzed using PLINK. All models were adjusted for covariates of components 1 and 2 from either principal components or multidimensional scaling analyses to account for population substructure and stochastic genotypic variation (except in the UK-GWAS data which were not adjusted for population substructure).

### Association test statistics

Single SNP test statistics were combined across datasets using a score test methodology, essentially assuming equal odds ratio across cohorts. In addition, fixed and random effects meta-analyses were implemented in R (version 2.11) to confirm that the score test approximation does not affect the interpretation of the results. We also tested the relevant SNPs heterogeneity across cohorts and no significant heterogeneity was detected (Table S2).

### Data exchange

We communicated to our colleagues in charge of the independent study (Do et al) the seven SNPs listed in Table 2. For this subset of SNPs they selected the marker with the highest r^2^ value on their genotyping platform and provided us with the following summary statistics: odds ratio, direction of effect, standard error for the estimated odds ratio and one degree-of-freedom trend test P-value.

### eQTL analysis and methylation analysis

Quantitative trait analyses were conducted to infer effects of risk SNPs on proximal CpG methylation and gene expression. For the five replicated SNP associations (Table 2), all available CpG probes and expression probes within +/−1 MB of the target SNP were investigated as candidate QTL associations in frontal cortex and cerebellar tissue samples. 399 samples were assayed for genome-wide gene expression on Illumina HumanHT-12 v3 Expression Beadchips and 292 samples were assayed using Infinium HumanMethylation27 Beadchips, both per manufacturer's protocols in each brain region. A more in depth description of the sample series comprising the QTL analyses, relevant laboratory procedures and quality requirements may be found in [15]. The QTL analysis utilized multivariate linear regression models to estimate effects of allele dosages per SNP on expression and methylation levels adjusted for covariates of age at death, gender, the first 2 component vectors from multi-dimensional scaling, post mortem interval (PMI), brain bank from where the samples were provided and in which preparation/hybridization batch the samples were processed. A total of 670 candidate QTL associations were tested: 87 expression QTLs in the cerebellum samples, 85 expression QTLs in the frontal cortex samples, 249 methylation QTLs in the cerebellum samples and 249 methylation QTLs in the frontal cortex samples. Multiple test correction was undertaken using false discovery rate adjusted p-values<0.05 to dictate significance, with the p-value adjustment undertaken in each series separately, stratified by brain region and assay. A complete list of all QTL associations tested is included in Table S3.

## Supporting Information

Table S1 Summary of results for fixed and random effects meta-analysis, as estimates of effect heterogeneity across cohorts and SNP used at the Do et al replication stage.
(XLSX)

Table S2 Summary of the quality control parameters applied to the GWA datasets included in this study.
(XLSX)

Table S3 Complete list of tested QTL associations (expression and methylation).
(XLSX)

Text S1 Membership of the Wellcome Trust Case Control Consortium 2.
(DOC)
